# Supplementary material for: Is the Intergenic Region of Aedes aegypti Totivirus a Recombination Hotspot?
Source: Viruses. 2022 Nov 8;14(11):2467. doi: 10.3390/v14112467 (PMC9699231; doi:10.3390/v14112467)
Supplement: Supplementary file 1 [file viruses-14-02467-s001.zip › viruses-1982831-supplementary.pdf]

Supplementary material.

Is the intergenic region of Aedes aegypti Totivirus a recombination hotspot?

Roseane da Silva Couto 1, Geovani de Oliveira Ribeiro1, Ramendra Pati Pandey 2 and Élcio Leal 1,*

1 Laboratório de Diversidade Viral, Instituto de Ciências Biológicas, Universidade Federal do Pará, Belem 66075-000, Bélem, Pará, Brazil
2 Centre for Drug Design Discovery and Development (C4D), SRM University, Delhi-NCR, Rajiv Gandhi Education City, Sonepat 131029, Haryana, India

* Correspondence: elcioleal@gmail.com

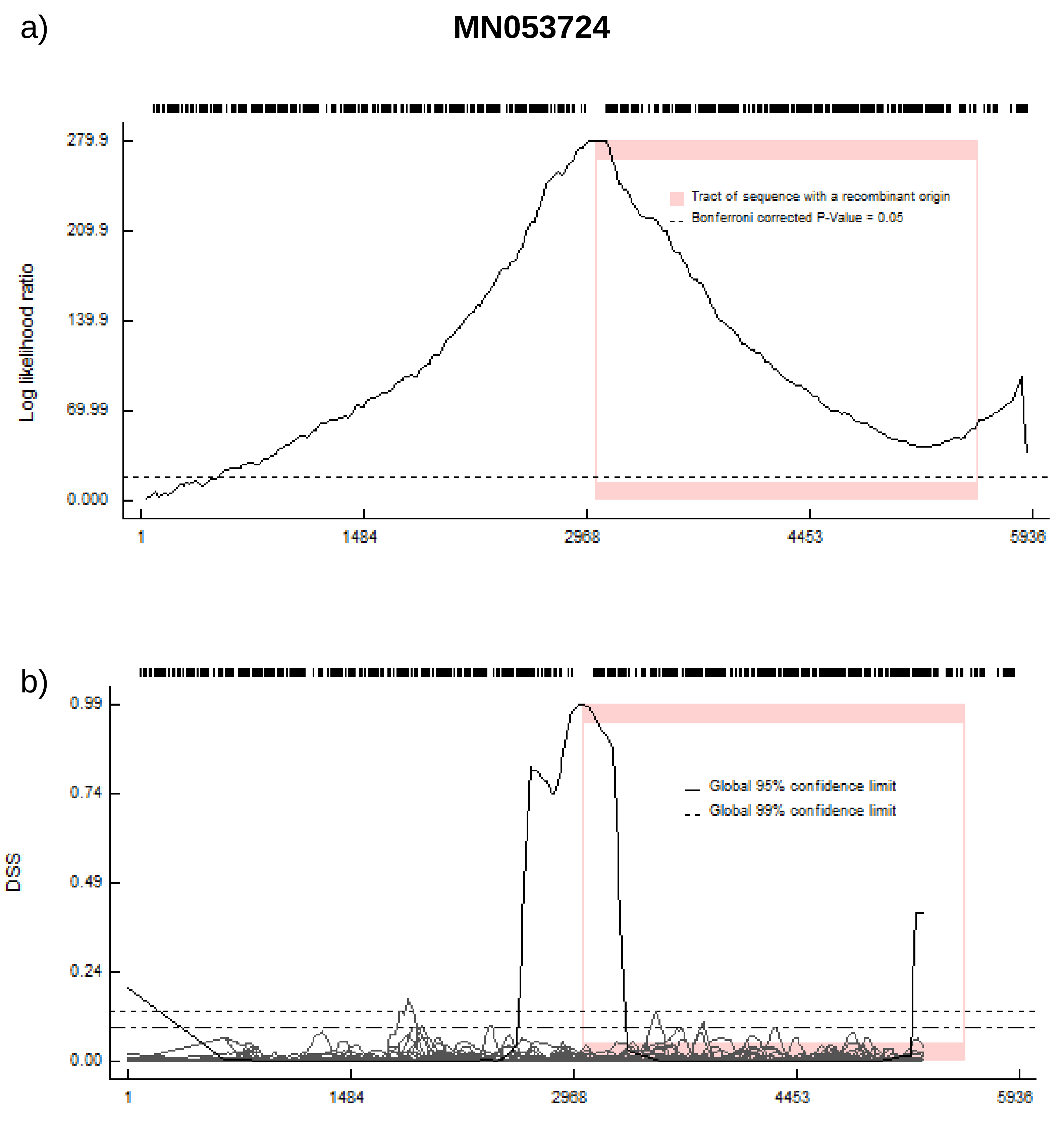

Figure S1. Recombination signal in AaTV MN053724. a) recombination signal detected by Lard in the genome of the sequence MN053724. The trace indicates a peek with high likelihood values around the nucleotide position 2968. b) Recombination signal detected by Topal (DSS) method. Ther DSS plot also indicates regions bounded by recombination breakpoints. Both methods converge to identify a single breakpoint in the MN053724 sequence. The pink area represents the point in the alignment that optimally separates regions of conflicting phylogenetic signal. Horizontal dark line indicates informative sites used in the analysis. Both methods search for differences in phylogenetic trees constructed from adjacent regions of sequence.

MN053725 and MN053732

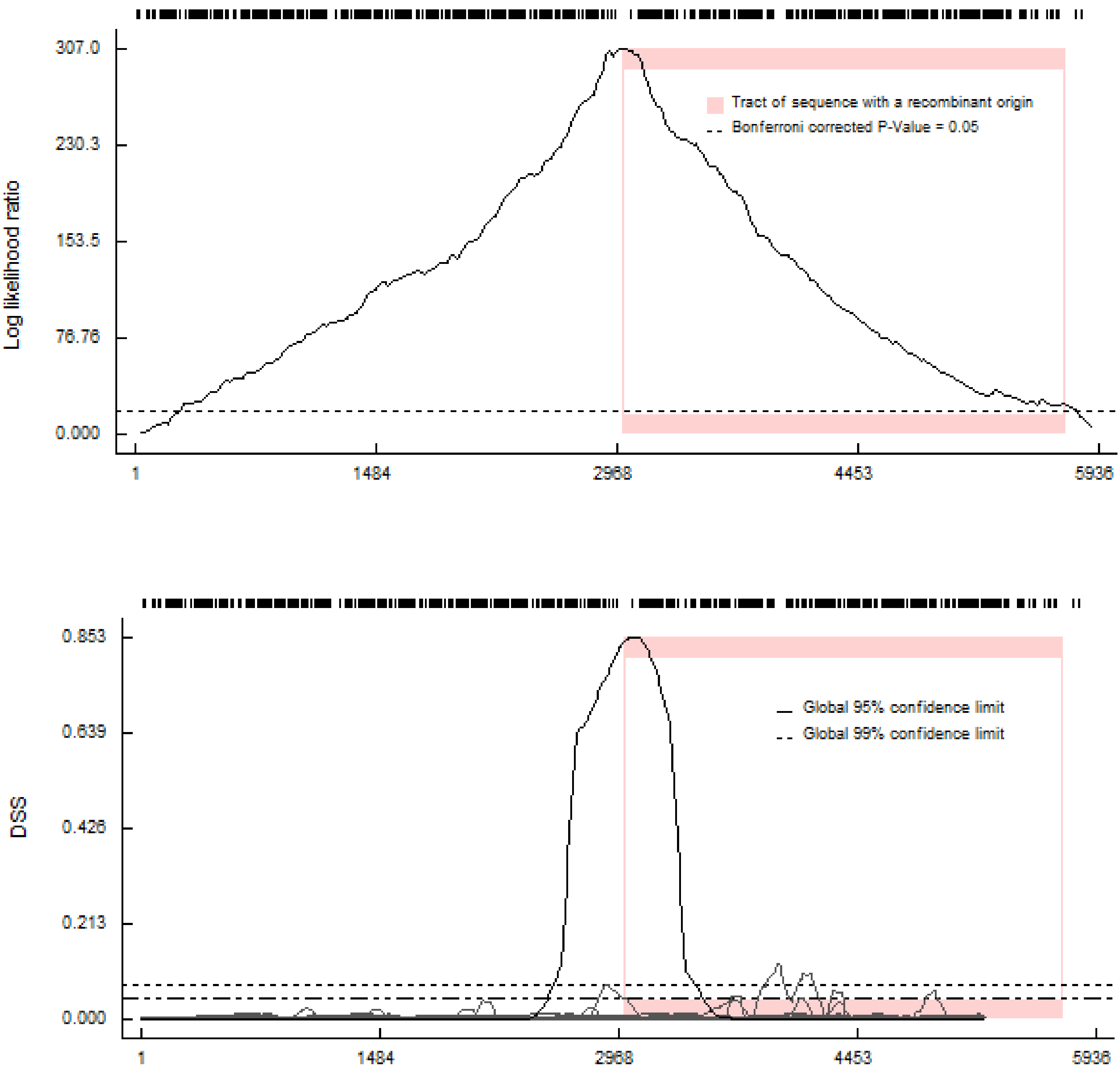

Figure S2. Recombination signal in AaTV MN053725 and MN053732. a) Recombination signal detected by Lard method in the sequence MN053724. The trace indicates a peek with highest likelihoods around the nucleotide position 2968. b) Recombination signal detected by Topal (DSS) method. The difference between the sum of squares (DSS) of the forced and unforced tree topologies is recorded for each partition. DSS peaks along the length of the alignment are indicative of potential recombination breakpoints. Both methods converge to identify a single breakpoint in MN053725 and MN053732 sequences. The pink area represents the point in the alignment that optimally separates regions of conflicting phylogenetic signal. Horizontal dark line indicates informative sites used in the analysis. Both methods search for differences in phylogenetic trees constructed from adjacent regions of sequence.

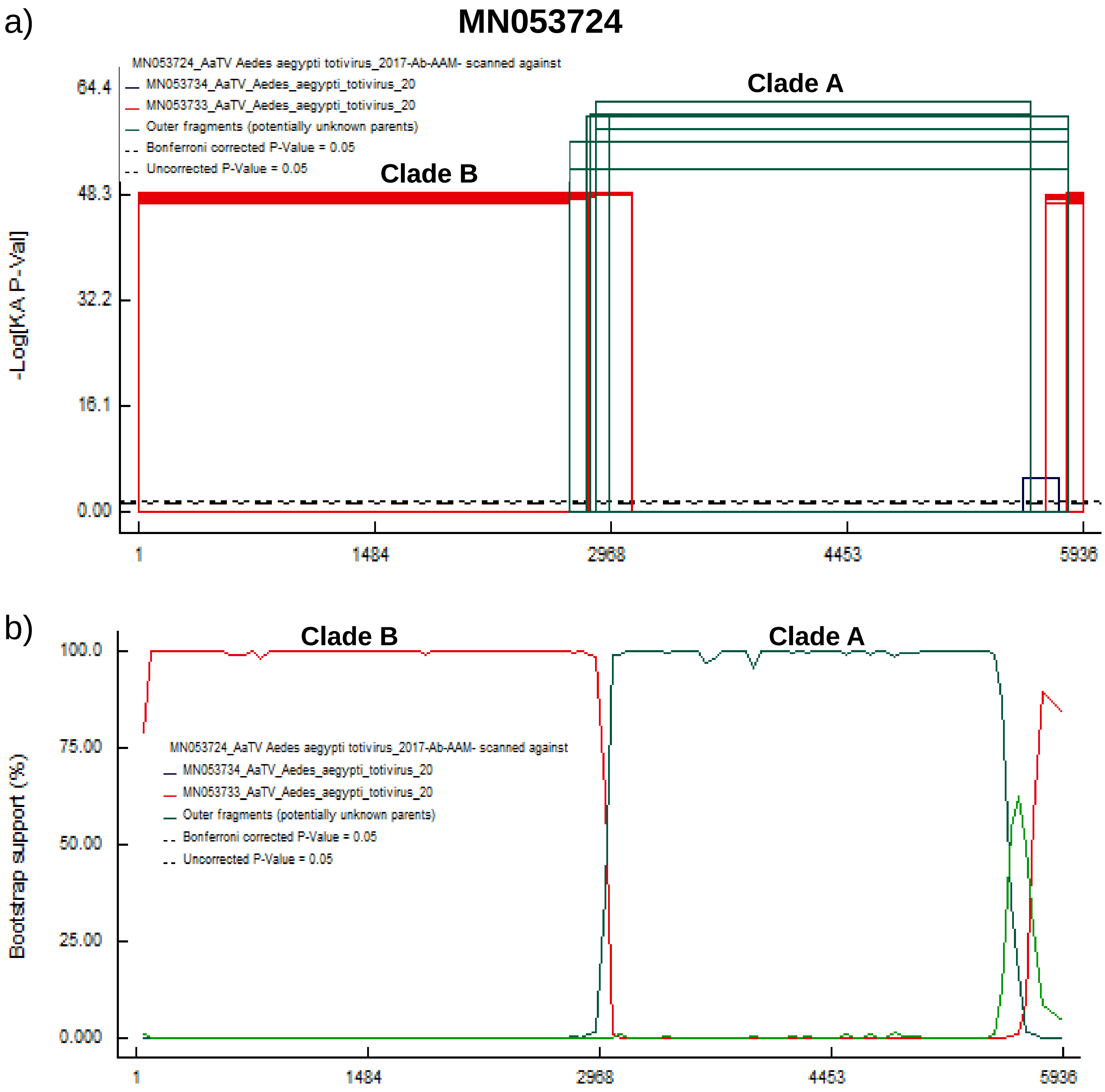

Figure S3. Mosaic Pattern of AaTV MN053724. a) Recombination plot obtained by the Geneconv method. The x-axis indicates the alignment position and y-axis indicates the high scoring similarities obtained by the corrected p-values (KA p-values). The red horizontal line is the region in the query (MN053724) with highest similarity with the parental sequence MN053733 from clade B and the green line is the region with highest similarity with MN053734 from clade A. b) Bootscanning plot. The y-axis indicates the bootstrap support of trees of trees obtained in different regions of the alignment. The red line is the bootstrap values of tree in which the query was grouped with the parental MN053733 (clade B) and the green line is the bootstrap values of tree grouping the query and MN05374 (clade A) parental sequence. Window size of 300bp and sliding windows of 50bp were used to draw this plot..

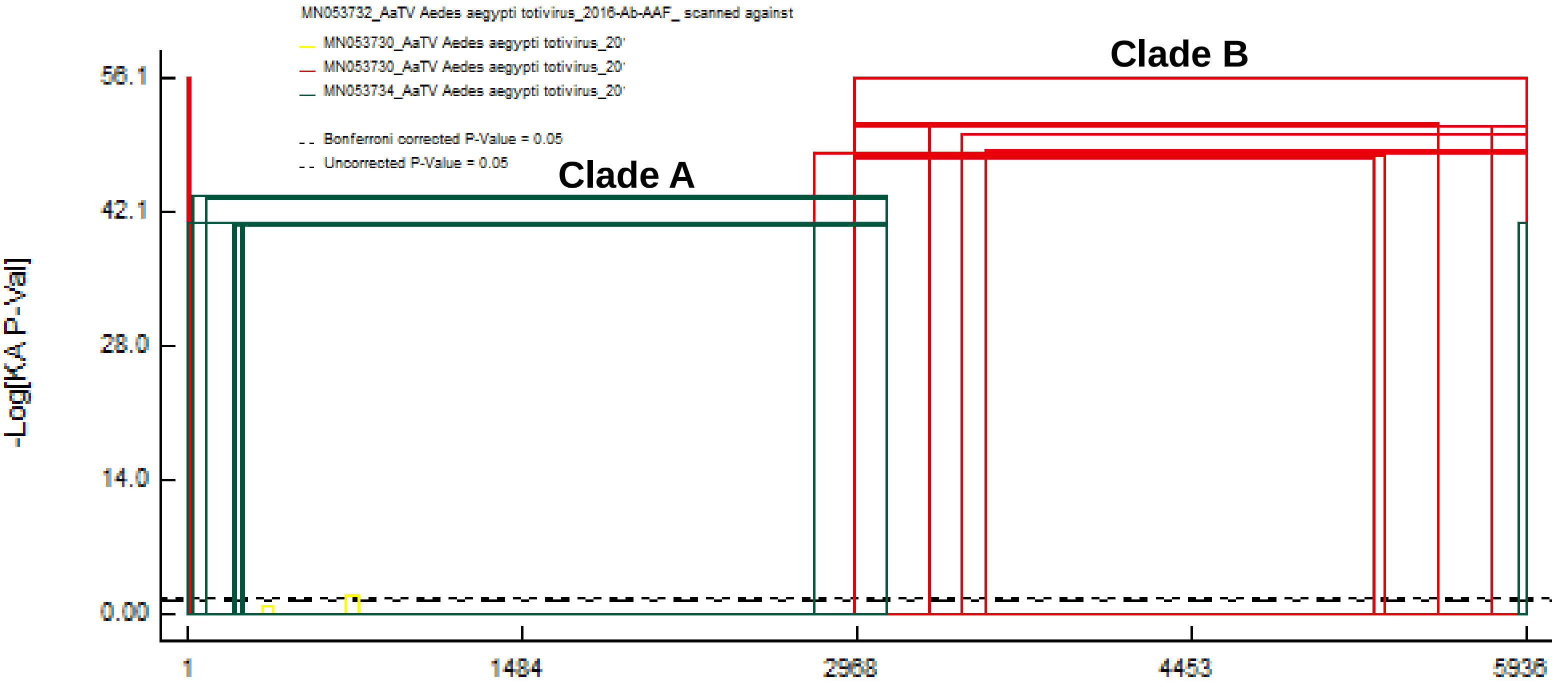

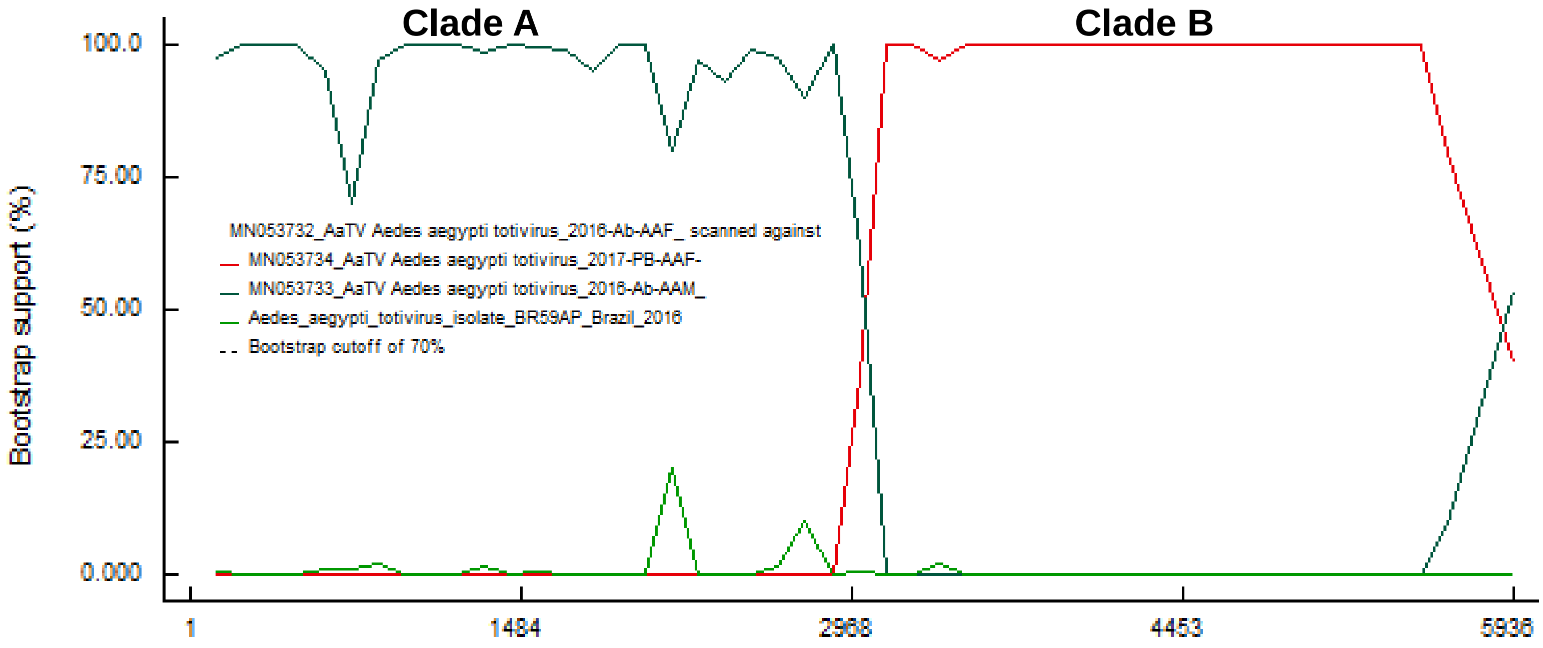

Figure S4. Mosaic Pattern of AaTV MN053725 and MN053732. a) Recombination plot obtained by the Geneconv method. The x-axis indicates the alignment position and y-axis indicates the high scoring similarities obtained by the corrected p-values (KA p-values). The red horizontal line is the region in the query sequence with highest similarity with the parental sequence MN053734 from clade A and the green line is the region with highest similarity with MN053733 from clade B. b) Bootscanning plot. The y-axis indicates the bootstrap support of trees of trees obtained in different regions of the alignment. The red line is the bootstrap scores of tree in which the query was grouped with the parental MN053734 (clade A) and the green line is the bootstrap values of tree grouping the query and MN053733 (clade B) parental sequence. Window size of 300bp and sliding windows of 50bp were used to draw this plot.
